# Estimating the distribution of a novel clinical biomarker (FGF-23) in the US population using findings from a regional research registry

**DOI:** 10.1371/journal.pone.0218435

**Published:** 2019-06-27

**Authors:** Joseph A. Johnston, David R. Nelson, Limin Zhang, Sarah E. Curtis, James R. Voelker, John R. Wetterau

**Affiliations:** 1 Global Patient Outcomes and Real World Evidence, Eli Lilly and Company, Indianapolis, Indiana, United States of America; 2 Lilly Research Laboratories, Eli Lilly and Company, Indianapolis, Indiana, United States of America; University of Colorado Denver School of Medicine, UNITED STATES

## Abstract

Evidence of involvement of novel biomarkers in disease pathogenesis from research cohorts often precedes an understanding of their distributions in broader populations. This study aimed to estimate the distribution of fibroblast growth factor 23 (FGF-23), an endocrine hormone that helps to regulate serum phosphate levels, in the overall US population and in important subgroups. We used a predictive model generated using data from the Framingham Health Study to estimate FGF-23 values for adults in the US National Health and Nutrition Examination Survey and the size of patient subgroups with levels of FGF-23 above selected thresholds. To assess the face validity of our FGF-23 estimates, we examined the relationship between estimated FGF-23 and cardiovascular and all-cause mortality within NHANES using Kaplan-Meier estimates and Cox proportional-hazards regression models and compared it to that observed in Framingham. Estimated FGF-23 values from NHANES were lower (median [interquartile range] 47.4 [35.8, 64.0] vs. 67.0 [54.0, 85.0] RU/mL) than the observed FGF-23 values from the Framingham cohort. Age- and sex-adjusted 10-year all-cause mortality was significantly higher (hazard ratio 2.43 [95% confidence interval: 1.42, 4.16]) for subjects with estimated FGF-23 levels in the highest versus lowest quartile. Estimating the distribution of biomarker values in the general population by applying predictive equations from smaller research cohorts is feasible and can inform drug research decision making.

## Introduction

The development of new medicines is often aided by the exploration of novel laboratory biomarkers and the role that they play in disease pathogenesis. Such biomarkers can inform the drug development process in several ways. Early on, they provide an invaluable tool for scientists working to elucidate disease pathways, identify treatment targets, and test the effects of potential drug candidates in pre-clinical disease models. Later, as pharmacodynamic measures, they can enable demonstration of proof of concept in early phase clinical research studies, pending confirmation of efficacy on more clinically meaningful patient outcomes in large Phase 3 clinical trials [1]. Finally, provided sufficient information on biomarker epidemiology, they can be used to estimate the size of the potential patient population to target with the new therapy, project treatment benefit, and inform decisions as to whether this projected benefit justifies further research investment.

Evidence of the association between a novel biomarker and the unfolding of disease in select cohorts often precedes the understanding of the prevalence of the biomarker in broader populations in which it may ultimately be used. For example, while findings from the Framingham Health Study (FHS) (i.e., approximately 5,000 adults living in Framingham, Massachusetts) provided compelling evidence that elevated cholesterol was associated with coronary heart disease as early as 1961 [2], an assessment of the applicability of findings from this study to the general US population remained a topic of debate more than 15 years later [3, 4], and the population epidemiology of hyperlipidemia continues to motivate publications to the present decade [5, 6]. In this example, as is frequently the case, stronger evidence of a causal link emerged much later with development of effective lipid lowering treatments.

Given this time lag in understanding biomarker epidemiology, drug development decisions are often made based on incomplete information about the extent to which abnormalities seen in small cohort studies translate to the overall disease population. With the growing availability of large, “real-world” datasets and the increasing potential for linkage between datasets with disparate data elements, the potential exists to leverage linked data to produce estimates of biomarker prevalence and hence the size of a potential disease population to target with a novel treatment directed at a specific mechanism reflected by that biomarker. In addition, such estimates can be used to partially validate biomarker-disease associations seen in convenience samples using larger datasets.

In the present study, we demonstrate this approach using the example of fibroblast growth factor 23 (FGF-23), an endocrine hormone primarily produced in bone that helps regulate serum phosphate levels by inducing renal phosphate excretion via effects in the proximal tubule and decreasing intestinal phosphate absorption [7, 8]. In patients with chronic kidney disease (CKD), serum FGF-23 levels increase as compensatory mechanisms fail, even before serum phosphate levels rise [9]. In addition to its salutary effects, there is growing evidence that FGF-23 has a direct and deleterious effect on the myocardium, leading to cardiac hypertrophy, cardiovascular disease (CVD) and increased mortality [10–14].

At present, as with cholesterol in the early 1960’s, associative but not causal evidence exists relating serum FGF-23 to cardiac disease in humans, and little is known about the distribution of serum FGF-23 levels in patients who might be especially responsive to a medication aimed at lowering or blocking the pathophysiologic effects of FGF-23, such as those with CKD or congestive heart failure (CHF). Indeed, even research cohorts defined based on specific clinical characteristics (e.g., stage 5 CKD) are generally not representative samples of the subgroups from which they are drawn. As a result, discretion is required when generalizing findings from these studies to more general populations.

To bridge this gap, we use a predictive model generated using data from the FHS to impute serum C-terminal FGF-23 (cFGF-23) levels for adults in the US National Health and Nutrition Examination Survey (NHANES), and estimate the size of various patient subgroups with levels of cFGF-23 above selected thresholds. In addition, to examine whether a population using estimated FGF-23 values exhibits results congruent to studies with actual FGF-23 samples, we explore the association between estimated cFGF-23 levels (referred to throughout as eFGF-23) and mortality using the NHANES-provided linkage to mortality data from the National Death Index. Our goal is to provide a case study demonstrating the utility of this approach.

## Methods

### Data sources

This is a retrospective observational study using publicly available, de-identified patient-level data from the US NHANES. NHANES is an ongoing program of studies conducted by the National Center for Health Statistics and the Centers for Disease Control and Prevention to assess the health and nutritional status of adults and children in the US [15]. NHANES employs a survey-weighted design, allowing estimates to be projected to the full non-institutionalized US population. Data from multiple 2-year cycles of continuous NHANES (1999–2014) were combined to increase robustness of estimates. While NHANES data are predominantly cross-sectional in nature, they have also been linked to the National Death Index and hence can be used to assess the relationship of various measurements to mortality, including ICD-10 coded specific cause of death [16]. Detailed descriptions of the design and data collection of NHANES have been published previously [17].

### Study populations

We included all adult NHANES participants (1999–2014) aged ≥20 years in the study population. Subgroups of interest included participants with CKD, identified by laboratory measurements, and those with CHF, identified through patient report. Estimated glomerular filtration rate (GFR) and urinary albumin-creation ratio were used to determine CKD stage in accordance with the Kidney Disease: Improving Global Outcomes (KDIGO) 2012 clinical practice guideline for the evaluation and management of CKD: stage 0, mildly decreased or high GFR (≥60 mL/min/1.73 m^2^) without albuminuria; stage 1, kidney damage with normal or increased GFR (≥90 mL/min/1.73 m^2^) and albuminuria; stage 2, kidney damage with mildly decreased GFR (60–89 mL/min/1.73 m^2^) and albuminuria; stage 3, moderately decreased GFR (30–59 mL/min/1.73 m^2^); stage 4, severely decreased GFR (15–29 mL/min/1.73 m^2^); stage 5, kidney failure (GFR <15 mL/min/1.73 m^2^ or dialysis) [18]. We used a urinary albumin-creatinine ratio of >30 mg/g, based on a single measurement of urine albumin and creatinine, as evidence of kidney damage, and calculated estimated GFR using a single assessment of serum creatinine and the Chronic Kidney Disease Epidemiology Collaboration equation [19]. Participants with CHF were defined as those who answered “yes” to the question: “Has a doctor or other health professional ever told you that you had congestive heart failure?” Participants with no kidney disease (GFR ≥60 with no albuminuria) and without CHF were used as reference groups. Only the NHANES fasting cohort was included and the fasting observation weighting utilized because certain key variables (e.g., fasting glucose) were recorded only among this cohort.

### Variables and measures

To date, no NHANES cohort has included FGF-23. In order to estimate FGF-23, we used a regression equation from Haring and colleagues [20]. This model was created using data from the FHS. Specifically, Haring and colleagues used data from a cohort of 3,236 individuals drawn from the seventh examination cycle (1998–2001) of the Framingham Offspring Study (n = 2,846) and the second examination cycle (1999–2001) from the Framingham Omni Study (n = 390) with complete FGF-23 and covariate data. The Framingham Offspring Study includes adult children of the original FHS cohort [21, 22], while the FHS Omni cohort includes an oversampling of ethnic minorities not well represented in the original FHS and FHS Offspring cohorts [23]. Variables used by Haring and colleagues in generating the predictive model for FGF-23 included age, sex, ethnicity, antihypertensive medication use, fasting serum glucose, history of CVD, antilipidemic medication use, current smoking, hormone replacement therapy use, waist circumference and estimated GFR, all of which are also available in NHANES. Data from surveys, physical examinations, and laboratory assessments of individuals participating in continuous NHANES were used to measure predictors of FGF-23 and stratification variables. Linkage of NHANES to National Death Index (NDI) data was used to assess mortality for 1999–2010 NHANES because mortality data was available through 2011. Linkage was conducted by the National Center for Health Statistics (NCHS) data linkage team. Approval for the linkage was provided by NCHS’ Research Ethics Review Board (ERB) and the linkage was performed only for eligible NCHS survey participants. Cardiovascular (CV) mortality was defined as death with “Diseases of heart” or “Cerebrovascular diseases” listed as the cause of death on the death record.

### Scaling the Framingham FGF-23 equation using an NHANES calibration subgroup

Haring and colleagues provided an equation to estimate FGF-23 based on their Framingham subgroup, the median and interquartile range (IQR) for the FGF-23 distribution in this cohort, and the summary statistics of key demographic and clinical measures. However, the published equation does not include an intercept. Therefore, our approach was to include all of the information available to implement the equation, without an intercept, in an NHANES sub-sample that was similar to the Framingham sample, and then rescale to match the FHS sample’s FGF-23 summary statistics, hence estimating the intercept. The Haring FGF-23 equation utilizes standardized independent variables and standardized dependent variables of a natural log transformed FGF-23. In order to ensure that FGF-23 estimates in NHANES were on the same scale as the Framingham measurements, we identified a subgroup of NHANES subjects comparable to the Framingham cohort and calibrated estimates such that the distribution of eFGF-23 for this subgroup matched the observed distribution of FGF-23 in the Framingham cohort.

To create this calibration subgroup, initially the NHANES age group of 43 years and above provided a subset with an age distribution similar to the Framingham cohort’s 58.8 (mean) and 11.2 (standard deviation). Next, the Framingham rate of 88.0% white was much higher than the rate of NHANES, so NHANES race and ethnicities were re-weighted to match the Framingham group. Lastly, even within this age and race/ethnicity matched subgroup, the published mean eGFR for Framingham subjects was lower than the NHANES mean. Differences in eGFR between NHANES and Framingham likely represent true differences in eGFR of the populations and not measurement variation. Therefore, only for the purposes of creating an FHS-type sample of NHANES, the NHANES subgroup’s eGFR values were “inflated” to have the same mean and standard deviation as those from Framingham. The resulting standardized ln(eFGF-23) was transformed so that the median and IQR was (67.0 RU/mL; 54.0, 85.0) within this subgroup of NHANES, equivalent to the FGF-23 results reported by Haring et al., hence addressing the problem of the missing intercept. For calculation of the NHANES estimates of eFGF-23, the actual eGFR measures were used, not the values transformed to match FHS.

### Statistical analyses

In order to understand the magnitude of differences between the source (FHS), target (overall NHANES), and calibration cohorts, we examined the demographic and laboratory characteristics of these cohorts side-by-side. We also compared (visually) the distribution of eFGF-23 levels for the NHANES cohort with the observed FGF-23 levels reported for the Framingham cohort. Distributions were also extended to subpopulations of interest within NHANES with various CKD stages, CHF, and combinations of both.

In order to produce unbiased national estimates of the prevalence of elevated FGF-23 overall and within subgroups of interest, we utilized sample weights (in addition to strata and clusters for proper standard error estimation) from NHANES within SAS survey procedures. To assess the face validity of our eFGF-23 estimates, we used Kaplan-Meier estimates and Cox proportional-hazards regression models to examine the age- and sex-adjusted relationship between eFGF-23 and CV and all-cause mortality in NHANES and compared these estimates to those from comparable models reported by Haring et al. [20]. Finally, to explore trade-offs between potential individual and population level benefits, we calculated the number needed to treat (NNT) required to avoid one all-cause death for a variety of NHANES patient subgroups of interest. For this purpose, we assumed a hypothetical treatment that would eliminate the attributable risk associated with elevated FGF-23 by reducing it from the median value for a given subgroup to a baseline value of 18 RU/ml, the lowest risk category used as a reference case in a recent meta-analysis by Qin and colleagues [24]. This was done by: (1) using the median eFGF-23 levels for our NHANES subgroups to visually estimate the corresponding relative risk for all-cause death shown in Fig 6 from the Qin study; (2) multiplying the baseline risk of 0.0325 by these relative risks to estimate the mortality risk for each subgroup (MR_sub_); and (3) calculating the corresponding NNTs using the equation NNT = 1/ (MR_sub_−0.0325).

All statistical analyses were conducted using SAS 9.4 software (SAS Institute, Cary, NC, USA).

## Results

Subjects in the Framingham cohort tended to be older (mean age 58.8 vs. 46.2 years), less ethnically diverse (12% vs. 30.2% non-white), and had a higher prevalence of hypertension (45.7% vs. 30.1%), type 2 diabetes (11.2% vs. 7.3%) and CVD (12.4% vs. 8.1%) than the adult US population estimated from the target NHANES group (Table 1). Framingham subjects also evidenced greater hormone replacement therapy use (18.6% vs. 9.7%) and lower eGFR (mean eGFR 70.6 vs. 95.4 mL/min) relative to NHANES. While the calibration procedure effectively minimized these differences, several differences remained, including higher rates of antilipidemic medication use (25.3% vs. 19.8%) and current smoking (17.2% vs. 13.0%) in the NHANES calibration subgroup; other differences appeared negligible.

**Table 1:** Comparison of framingham, NHANES calibration subgroup used for scaling eFGF-23, and overall NHANES samples.

Characteristic	Framingham (*n* = 3,236)	NHANES Calibration Subgroup (*n* = 112,401,814)^‡^	Overall NHANES (*n* = 203,780,775)^‡^
Age,*^†^ years	58.8 (11.2)	58.9 (11.3)	46.2 (16.6)
Male sex,* %	45.6	47.7	48.4
Ethnicity,*^†^ %			
White	88.0	88.0	69.8
African American	4.8	4.8	10.9
Hispanic	4.2	4.2	13.3
Asian	3.0	n/a	n/a
Native American	0.1	n/a	n/a
Other	n/a	3.0	6.0
Hypertension, %	45.7	43.1	30.1
Antihypertensive medication,* %	32.8	34.8	21.5
Type 2 diabetes mellitus, %	11.2	10.7	7.3
Fasting serum glucose,* mg/dL	104 (30)	107.8 (30.1)	103.1 (28.6)
History of CVD,* %	12.4	13.7	8.1
Total cholesterol, mg/dL	200 (37)	204.4 (42.3)	196.8 (41.6)
HDL cholesterol, mg/dL	54 (17)	54.6 (16.4)	53.3 (15.7)
LDL cholesterol, mg/dL	120 (34)	121.3 (36.0)	117.0 (35.2)
Antilipidemic medication, %	19.8	25.3	14.7
Current smoker,* %	13.0	17.2	20.1
Hormone replacement therapy,* %	18.6	17.7	9.7
Waist circumference,* cm	99.6 (14.5)	100.6 (15.3)	97.7 (16.1)
BMI, kg/m^2^	28.1 (5.4)	28.9 (6.3)	28.5 (6.5)
eGFR,*^†^ mL/min per 1.73 m^2^	70.6 (16.3)	70.6 (16.3)	95.4 (22.1)

Abbreviations: BMI, body mass index; CVD, cardiovascular disease; eFGF-23, estimated fibroblast growth factor 23; eGFR, estimated glomerular filtration rate; HDL, high-density lipoprotein; LDL, low-density lipoprotein; n/a = not applicable; NHANES, National Health and Nutrition Examination Survey; SD, standard deviation.

Data are percentages and mean (SD).

*Variables included in predictive modeling by Haring et al. [20] and used for estimating FGF-23 levels in NHANES.

^†^Variables used in calibrating eFGF.

^‡^Sample sizes, values, and percentages in these columns reflect projected population after application of NHANES survey weights to corresponding NHANES samples. Weighted n’s for specific characteristics are lower when missing values are present.

**Fig 1:**
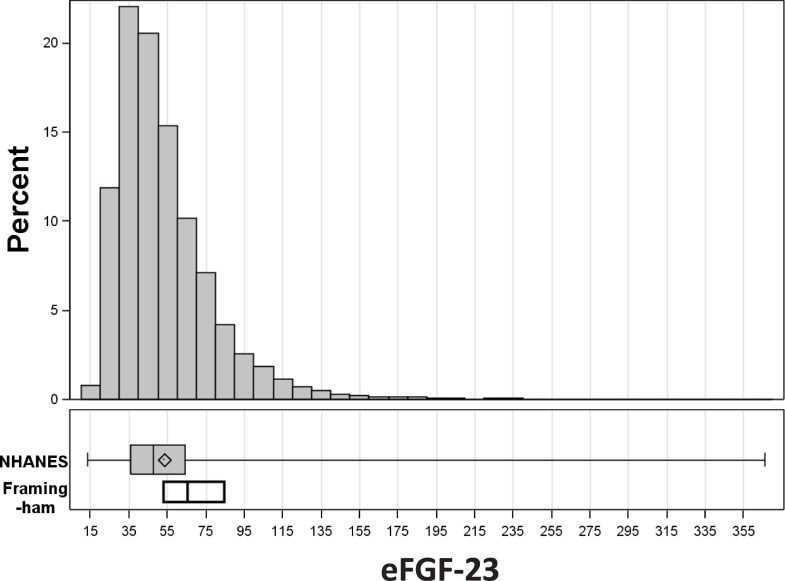
Comparison of the distributions of eFGF-23 from NHANES and observed FGF-23 from Framingham. The top portion of the figure shows the eFGF-23 frequency distribution for NHANES subjects. The boxplots in the lower portion show the quartiles for eFGF-23 from NHANES and the corresponding observed median and IQR FGF-23 values from Framingham, as reported by Haring and colleagues. Adapted from J Am Heart Assoc. 2016;5: e003486.

**Fig 2:**
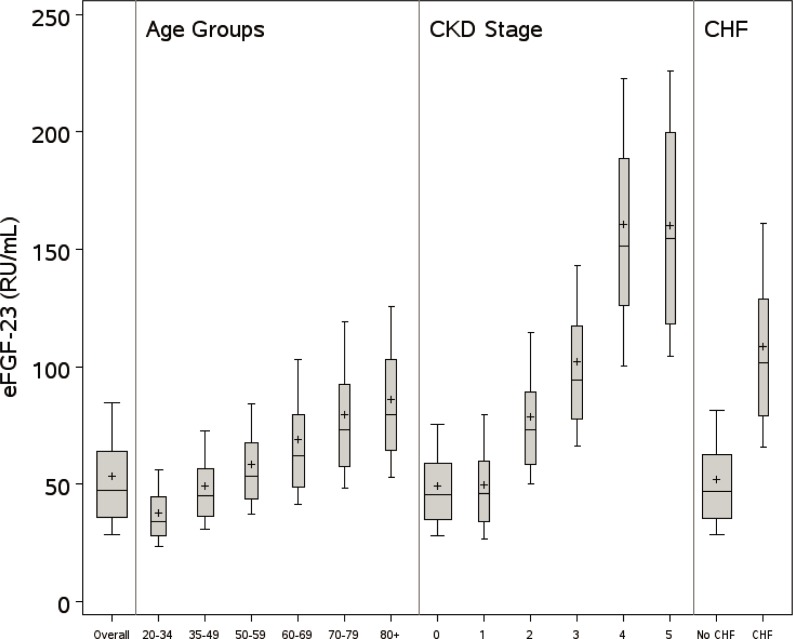
Distribution of eFGF-23 among subgroups. Boxplots show median, IQR, and 95% CI for eFGF-23 for NHANES subjects overall and for subgroups defined by age, CKD stage, and presence of CHF within the categories indicated on the x-axis. Plus symbols in boxplots designate means of distributions.

The eFGF-23 values from the overall weighted NHANES population were lower (median [IQR] 47.4 [35.8, 64.0] vs. 67.0 [54.0, 85.0] RU/mL) than the only published FGF-23 percentiles from the Framingham cohort (Fig 1), most notably due to the older age and lower eGFR − both key components of the Haring equation [20] − among FHS participants. Mean eFGF-23 levels in the NHANES population increased steadily with age and CKD, with marked increases noted among subjects with CKD stage 4 and 5 (Fig 2). Mean eFGF-23 was more than twice as high among those with versus without a self-reported history of CHF.

Using different thresholds for what constitutes an “elevated” FGF-23 level resulted in different population size estimates. For example, an estimated 79.1%, 46.6% and 19.7% of NHANES subjects aged 60 to 69 have eFGF-23 levels above the 50^th^ (47.4 RU/ml), 75^th^ (64.0 RU/ml) and 90^th^ (84.4 RU/ml) percentiles of the overall NHANES distribution, respectively. Similarly, nearly all (93.0%) NHANES subjects with stage 2 CKD have an eFGF-23 above the NHANES median, whereas 66.2% and 30.3% have values above the 75^th^ and 90^th^ percentiles. Among subjects with CHF, nearly all (96.2%) are above the NHANES median and about two-thirds (67.8%) have values above the 90^th^ percentile.

**Fig 3:**
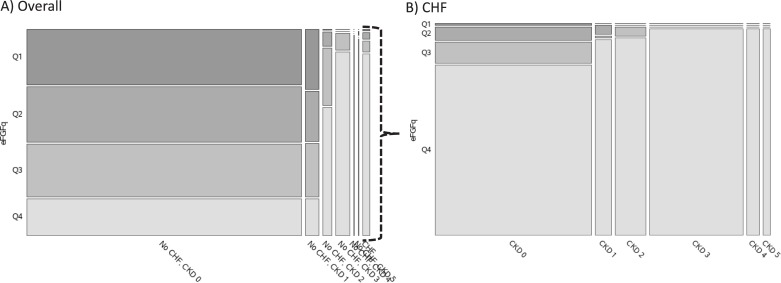
Size of NHANES subgroups by quartile of eFGF-23, CKD stage, and CHF status. The area of each tile in the mosaic plots is proportional to the number of subjects with the corresponding CKD stage and CHF status (x axis) and eFGF-23 in the indicated quartile from the overall NHANES cohort (y axis). The plot on the right further subdivides subjects with CHF.

A different pattern emerges when considering which subgroups contribute most to the overall population of subjects with “elevated” eFGF-23 levels, as seen in Fig 3. For example, among the 25% of NHANES subjects with an eFGF-23 value in the highest quartile (≥63.8 RU/mL), the 2 largest constituent subgroups are (i) subjects with normal renal function and no CHF, and (ii) subjects with CKD3 and no CHF. A similar pattern holds for subgroups within the subset of subjects with CHF.

**Fig 4:**
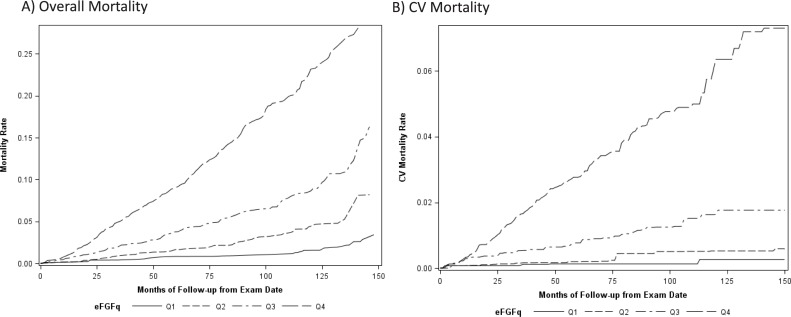
Kaplan-Meier estimates of overall and CV mortality among NHANES subjects by strata of eFGF-23. Estimated overall (left panel) and CV (right panel) mortality are shown on the y-axis by month since exam date on the x-axis for NHANES subgroups defined by quartile of eFGF-23. Estimates are adjusted for age, gender, and race/ethnicity.

The overall and CV mortality rates associated with eFGF-23 in the NHANES population are shown by quartile in Fig 4. Over a period of 10 years, Kaplan-Meier methods estimate 501,824 (1.6%) subjects with eFGF-23 levels in the lowest quartile died, 84,513 (0.3%) from CV causes. In contrast, 6,527,151 (23.2%) with eFGF-23 levels in the highest quartile died, 1,714,723 (6.4%) from CV causes.

The association between the continuous measure of eFGF-23 and estimated 10-year mortality was also significant in adjusted Cox proportional-hazards models (Table 2). In our models using NHANES data, there was a statistically significant (per SD) increase in estimated 10-year CV and all-cause mortality before and after adjustment for age and sex, similar to what was observed by Haring and colleagues. In contrast, while directionally similar, the observed hazard ratios when comparing subjects in the highest versus lowest quartile of eFGF-23 was statistically significant only for all-cause and not CV mortality.

**Table 2:** Associations between observed FGF-23 (Framingham), estimated FGF-23 (NHANES), and 10-year cardiovascular and all-cause mortality.

Fibroblast Growth Factor 23	Framingham (Haring et al. [20])		NHANES (1999–2010)	
	HR	95% CI	HR	95% CI
CV mortality*				
Age- and sex-adjusted model				
Continuous (per SD increase)	**1.53**	**1.29, 1.82**	**1.35**	**1.27, 1.45**
Q1	Ref.		Ref.	
Q2	0.44	0.19, 1.03	0.78	0.21, 2.91
Q3	1.00	0.52, 1.95	1.54	0.45, 5.29
Q4	**2.08**	**1.17, 3.69**	3.39	0.97, 11.92
All-cause mortality				
Age- and sex-adjusted model				
Continuous (per SD increase)	**1.42**	**1.30, 1.54**	**1.24**	**1.18, 1.29**
Q1	Ref.		Ref.	
Q2	1.23	0.88, 1.72	0.99	0.60, 1.62
Q3	1.27	0.91, 1.76	1.47	0.88, 2.47
Q4	**2.27**	**1.69, 3.05**	**2.43**	**1.42, 4.16**

Abbreviations: CI, confidence interval; CV, cardiovascular; FGF-23, fibroblast growth factor 23; HR, hazard ratio; NDI, National Death Index; NHANES, National Health and Nutrition Examination Survey; Q1, quartile 1; Q2, quartile 2; Q3, quartile 3; Q4, quartile 4; Ref, reference; SD, standard deviation.

*CV deaths in Framingham were clinically adjudicated over a median follow-up of 10.8 years [23], while CV mortality in NHANES was assessed based on cause of death reported on NDI death records.

The potential population and individual level benefits from a hypothetical FGF-23-lowering agent are shown in Table 3. In general, the NNTs were lower for smaller subgroups with higher average mortality risks than for larger groups. For example, the NNT for the 0.5% of individuals with stage 4+ CKD is lower (18) than the NNT for the 5.7% of those with stage 3+ CKD (32). In contrast, two groups of different sizes, those with stage 3+ CKD and those with CHF had similar baseline mortality risks and hence similar NNTs.

**Table 3:** Estimated US population prevalence and number needed to treat to avoid all-cause mortality in select NHANES patient subgroups.

NHANES Subgroup	Estimated US Population Prevalence	eFGF-23 (median, RU/ml)	Baseline Mortality Risk	NNT to Avoid All-Cause Death^†^
Reference*		18	3.3%	‒
eFGF23 Quartile				
Q3+	50%	64	4.6%	77
Q4 only	25%	79	5.5%	44
CKD				
Stage 3+	5.7%	97	6.4%	32
Stage 4+	0.5%	154	9.1%	18
Stage 5	0.2%	155	9.1%	18
CHF				
Yes	2.2%	102	6.4%	32

Abbreviations: NHANES, National Health and Nutrition Examination Survey; US, United States; eFGF-23, estimated fibroblast growth factor 23; IQR, interquartile range; NNT, number needed to treat; CKD, chronic kidney disease; CHF, congestive heart failure; Q3, quartile 3; Q4, quartile 4.

*The lowest C-terminal FGF-23 concentration (18 RU/ml) from the seven studies included in meta-analysis by Qin and colleagues [34] was used as a reference for calculating mortality risk reductions.

^†^NNT values were calculated using median subgroup eFGF-23 levels and relative risk reduction based on a recent meta-analysis from Qin and colleagues.

## Discussion

In this study, we demonstrated an approach to using a published predictive model from Framingham to estimate FGF-23 values for all adult subjects in NHANES and, by applying NHANES sample weights, estimate the distribution of FGF-23 levels in the overall adult US population. The viability and utility of this approach depends on the degree of overlap between the source and target populations: a certain degree of overlap is required to support the projection. If too similar, estimation would be unnecessary, while insufficient overlap leads to excessive uncertainty due to extrapolation. In our case, the source population (i.e., the Framingham cohort) was older and had a higher burden of CV disease than the target NHANES population, yet was comparable to a calibration cohort comprised of more than half of all adult subjects in NHANES. This situation may be more optimal relative to the typical situation in which early understanding of a novel biomarker is derived from smaller niche research cohorts with a disease process of interest. Establishing appropriate bounds on the use of this type of approach requires replication in scenarios with different degrees of source-target population overlap, and ultimately validation through direct measurement of biomarker values in epidemiologic cohorts such as NHANES.

Perhaps the most salient finding relative to eFGF-23 from this study is the discrepancy between the groups with the highest values and those with the greatest number of subjects with elevated values. Mean eFGF-23 levels increased steadily with age and CKD, and were more than twice as high among those with versus without CHF. However, the subgroups with the largest absolute numbers of subjects in the top quartile of eFGF-23 were those with earlier stages of CKD and without CHF. This highlights a challenge faced in developing new therapeutics targeting FGF-23, particularly during late clinical development. While targeting patients from smaller subgroups with the greatest absolute risk (e.g., those with end-stage CKD or CHF) may increase the likelihood of technical success for the therapy, a strategy aimed at treating larger subgroups with greater overall population burden of disease may facilitate patient enrollment, permit market entry at a lower price, and increase the likelihood of commercial success. The absolute risk reduction would not be as high and thus the number needed to treat to prevent an adverse disease outcome would be higher relative to that in the smaller subgroup patients at greater risk. This tension between population and individual level treatment benefit is clearly apparent in the NNT estimates in Table 3. Furthermore, identifying and recruiting patients without recognizable clinical disease presents its own challenges, and CKD is notoriously underdiagnosed despite inexpensive and readily available biochemical measures (i.e., serum creatinine and albumin-creatinine ratio) to make the diagnosis. For example, in a cohort of health-seeking ambulatory patients in Rochester NY, Ryan and colleagues found that only 26.5% of patients with laboratory evidence of CKD and 14.1% of those with stage 3 disease had been clinically diagnosed based on chart review [25].

The role of FGF-23 in disease has been extensively studied in the context of CKD. In CKD, the ability to balance the intake of dietary phosphate with the loss of phosphate through excretion by the kidney is impaired. The resultant increase in the body’s phosphate burden produces an increase in FGF-23 levels. The elevated FGF-23 may reflect tubular dysfunction and resistance to endocrine factors in kidney disease, an important pathology not fully captured by measurement of GFR or albuminuria [26].

High levels of FGF-23 appear to have a causal role in CVD. Elevated FGF-23 is more closely associated to left ventricular hypertrophy and heart failure than atherosclerosis related CVD [13, 27, 28]. In addition to promoting left ventricular hypertrophy [10], which can lead to arrhythmias and sudden cardiac death [29], other proposed direct pathological effects of FGF-23 include promoting endothelial dysfunction [30], pro-fibrotic effects [31], pro-inflammatory effects [32], and impaired immunity [33]. Alternatively, elevated FGF-23 may be an innocent bystander in disease, particularly at lower levels. For example, elevated phosphate burden or decreased klotho may directly promote CVD. Both changes also contribute to increased serum FGF-23 levels. Thus, it is possible that under some circumstances, elevated FGF-23 is merely a surrogate for changes in other factors (e.g., phosphate and klotho) more directly involved in the pathophysiology but by itself contributes little to the disease process.

At what concentration FGF-23 transitions from a bystander, risk associated factor, to a significant maladaptive, toxic, disease-causing factor in not clear [34]. Understanding this relationship between concentration and adverse effect has obvious therapeutic implications. The different assays currently used to quantitate FGF-23 levels, and the poor agreement between the various commercial assays hinders their clinical utility [35], as well as the practical utility of our findings relative to FGF-23. This is in part due to differences in assay calibration and the form of FGF-23 detected (i.e., intact FGF-23 versus cFGF-23). This calls for the field to develop more validated and uniform measures of FGF-23. This would greatly facilitate the application of FGF-23 as a biomarker for risk stratification, understanding disease severity, identifying the levels of a disease causing factor, and response to therapy following an intervention. This could provide an opportunity to more fully exploit the information contained in measures of FGF-23 in patients with CKD, CHF, and importantly, the many individuals with elevated FGF-23 in the non-CKD, non-CHF populations.

Among this study’s other limitations is that certain variables included in the regression equation for FGF-23 were defined or characterized differently in Framingham versus NHANES, which may have introduced bias into our estimates. For example, age in NHANES is truncated at 85, placing a ceiling on the contribution of age to eFGF-23. In addition, the Framingham smoking definition reflects more than a cigarette per day over the past year, whereas NHANES utilized a recent 30-day time frame.

Furthermore, in the absence of direct measurement of FGF-23 in at least a subset of NHANES patients, it is impossible to judge whether the Haring FGF-23 equation has external validity when applied to the more diverse NHANES population. The fact that the association between estimated NHANES FGF-23 values and CV and all-cause mortality was similar to what was observed by Haring and colleagues provides some support for the external validity of their equation. It is also interesting to note that the estimated FGF-23 levels for NHANES patients with CHF (not included in the Haring equation) were more than twice as high as among those without CHF, consistent with findings from other studies [36]. Nevertheless, the strength of our results hinge upon the strength of the assumption that the Haring equation can be validly extended to the broader NHANES population, an assumption that could best be tested were directly measured FGF-23 to be included as part of a future wave of NHANES.

A further limitation relates to the overlap in variables used to estimate FGF-23 and those used for risk adjustment in the mortality models. This could in part account for the decreased precision in the estimates of the associations between FGF-23 and CV and all-cause mortality in our models relative to what was observed by Haring and colleagues. Nevertheless, estimates from our models are directionally consistent with those from the Haring study, and most of the point estimates from Haring are contained within the confidence intervals from our study, as seen in Table 2.

Despite these limitations, we believe an estimate of the population distribution of biomarker values can inform research agendas and enhance drug research decision making. Data from analyses like these could be used to derive estimated numbers needed to treat (NNTs) to prevent a CV death among potential target subgroups at specific time intervals and assuming a variety of hypothetical efficacy levels (e.g., 10%, 20%, 30% reduction in eFGF-23). Researchers might then use insights gained not only to inform optimal target population selection but also to make value-based determinations of which programs to move forward or terminate.

In conclusion, it is feasible to estimate the distribution of novel biomarker values in the general population by applying predictive equations from smaller research cohorts to weighted national survey data. The resulting information can provide insights into the relative size of subgroups with elevated values, informing future research efforts. With respect to FGF-23, whereas older subjects with advanced CKD and CHF have the highest estimated values, targeting larger subgroups with less advanced CKD and without CHF may lead to provision of more meaningful public health benefits. Ultimately, the optimal choice of treatment targets requires carefully weighing anticipated patient and population level benefits.
